# Notch polymorphisms associated with sensitivity of noise induced hearing loss among Chinese textile factory workers

**DOI:** 10.1186/s12881-018-0676-8

**Published:** 2018-09-14

**Authors:** Enmin Ding, Jing Liu, Huanxi Shen, Wei Gong, Hengdong Zhang, Haiyan Song, Baoli Zhu

**Affiliations:** 1 Institute of Occupational Disease Prevention, Jiangsu Provincial Center for Disease Prevention and Control, No.172 Jiangsu Road, Nanjing, Jiangsu Province 210009 People’s Republic of China; 2 Nanjing Prevention and Treatment Center for Occupational Disease, Nanjing, Jiangsu Province China; 3 Kunshan Centers for Disease Prevention and Control, Kunshan, Jiangsu Province China

**Keywords:** NIHL, Notch1, Single nucleotide polymorphism, Haplotype

## Abstract

**Background:**

Noise induced hearing loss (NIHL) is a polygenic disease involving both genetic and environmental factors, and is one of the most important occupational health hazards worldwide. To date, the influence of Notch1 variants on the risk to develop NIHL has not been illuminated. This study was conducted to explore the effects of Notch1 polymorphisms on individual susceptibility to NIHL.

**Methods:**

A total of 2689 industrial workers from one textile factory in east China were recruited to participate in the current study. Venous blood was collected, basic clinical data was obtained by questionnaires and pure-tone audiometry (PTA) tests were conducted by specialist physicians. Next we performed genotyping of three selected SNPs (rs3124594, rs3124599 and rs3124603) in the Notch1 gene in 535 NIHL patients and 535 controls. Subsequently, the main effects of the genotypes and their interactions were evaluated.

**Results:**

Our results revealed that individuals with a GG of rs3124594, TT of rs3124603 (OR = 4.70 and 1.59 respectively) and the haplotype AAC (rs3124594-rs3124599-rs3124603) (OR = 14.95) were associated with an increased risk of NIHL in our study cohort. Stratified analysis showed that an increased NIHL risk was found in individuals exposed to work related noise for ≤16 years that also had the rs3124594 GG or rs3124603 CT/TT genotype with an OR of 4.20 and 1.73 respectively. Multifactor dimensionality reduction analysis indicated that rs3124594, rs3124599 and rs3124603 interacted with each other and were related to an increased risk to develop NIHL (OR = 3.60).

**Conclusions:**

The genetic polymorphisms rs3124594 and rs3124603 within the Notch1 gene are associated with an increased risk of NIHL in a Chinese population and could potentially be used as biomarkers for NIHL in noise exposed workers.

## Background

Occupational noise is one of the most common occupational hazards affecting the health of industrial workers, and noise-induced hearing loss (NIHL) has been the second most frequent form of sensorineural hearing loss after age-related hearing loss worldwide [1]. It has been widely acknowledged that NIHL is a multifactorial disease resulting from the interaction of genetic and environmental factors [2], but the mechanisms of NIHL are not well understood. It is thought that the etiopathogenesis may involve a direct mechanical injury to the structures of the cochlea, inner ear cell apoptosis and necrosis caused by oxidative stress, or metabolic products generated during signal transduction [3–5]. At present, numerous population-based inherited susceptibility studies have indicated that individuals exhibit variable degrees of NIHL susceptibility, even when exposed to equal noise intensity levels [1, 6]. Previous studies have found that single nucleotide polymorphisms (SNPs) in the *HSP70, EYA4, CDH23, GRHL2, FOXO3* and *DFNA5* genes are associated with genetic susceptibility to NIHL in humans, and may increase or decrease the risk of NIHL [7–10]. Studies using animal models have also shown that genetic variants contribute to the incidence of NIHL in mice [11, 12]. Therefore, it has been suggested that inherited susceptibility, environmental factors and their interactions may play vital roles in the occurrence and development of NIHL.

The Notch pathway is best known for its critical role in lateral inhibition, and is one of the key signaling pathways that regulate the development of the organ of Corti. It has been recognized that lateral inhibition controls the cell fate determination in the cochlea through Notch pathway regulation [13]. In the mouse, the Notch receptor Notch1 is expressed in the developing cochlea, including the presumptive sensory epithelium, while the Notch ligands Dll1 and Jag2 are expressed in the developing hair cells (HC) [14–16]. Loss of either Notch ligand Dll1 or Jag2 in the developing cochlea was found to result in the formation of extra rows of hair cells [17]. Furthermore, there is evidence that neonatal mammalian cochlear supporting cells have the ability to trans-differentiate into new hair cells when the Notch signaling pathway is blocked [18]. In a recent study, the application of gamma-secretase inhibitors that blocks the Notch pathway to noise-damaged cochleas was found to induce the generation of small numbers of new hair cells and led to a partial restoration of hearing, suggesting that the Notch pathway may still be active in the mature cochlea [19]. Furthermore, the loss of responsiveness to inhibition of the Notch pathway was partly induced by the downregulation of Notch receptors and ligands. This downregulation still persisted in the adult animals, even under conditions of noise damage [20]. Taken together, Notch signaling may play a vital role both in maintaining the homeostasis of the cochlear sensory epithelium and the mitotic regeneration of hair cells.

However, associations between noise induced hearing loss and Notch1 SNPs and their functional significance in the Notch1 gene on NIHL have not yet been studied. Literature review indicated that rs3124594 and rs3124599 of Notch1 were reported in previous studies regarding on lung cancer in northeast Chinese [21]. Even through Notch SNPs were mostly reported in cancer, considering the vital functions of Notch1 in hearing maintenance and repair, we hypothesized that polymorphisms in the Notch1 gene may also be associated with the genetic susceptibility to NIHL. Therefore, we conducted a case-control study to investigate the associations between three Notch1 SNPs, namely rs3124594, rs3124599 and rs3124603, with the genetic susceptibility of NIHL in noise exposed Chinese workers.

## Methods

### Subjects

The methods and study subjects have been described in our previous studies [10, 22]. Employees from a single textile factory in eastern China who received annual health examinations performed by the Jiangsu Provincial Center for Disease Prevention and Control were recruited for our study in 2015. A total of 2689 individuals participated in the health examinations. Informed consent was obtained from all participants at the beginning of the study. The study was approved by the ethical committee of the Jiangsu Provincial Center for Disease Prevention and Control. The occupational health examination included venous blood collection, a general physical examination and pure-tone audiometry (PTA). During the health examination, personal medical history and use of medical drugs as well as tobacco and alcohol consumption habits were assessed. Exclusion criteria were the presence of diseases that may affect the hearing thresholds (e.g. otitis media, cholesteatoma or ear canal stenosis) and current or former use of ototoxic drugs (e.g. aspirin, quinolones, and aminoglycosides). A total of 2477 subjects met our criteria and were included in the study.

### PTA and NIHL assessment

PTA was performed for each participant after a break from noise exposure for over 12 h. The audiometry was conducted by qualified physicians in a soundproof room using a Madsen Voyager 522 audiometer (Madsen, Taastrup, Denmark).

### Definitions of NIHL and control subjects

Hearing loss and normal hearing were defined according to the Chinese diagnostic criteria for occupational noise-induced deafness (GBZ 49–2007). In this study, occupational noise exposure was defined as levels of noise exposure (Lex) of at least 85 dB (A) during a nominal 8-h working day. Hearing loss was identified using binaural hearing thresholds that exceed 25 dB at both high (3000, 4000, 6000 Hz) and voice (500, 1000, 2000 Hz) frequencies. Normal hearing was identified by binaural hearing thresholds below 25 dB both at high and voice frequencies. Hearing thresholds were determined by PTA. The subjects were divided into two groups: NIHL patients (noise-exposed individuals with hearing loss) and control individuals (noise-exposed individuals with normal hearing). In total 535 NIHL patients were identified, and 535 controls matched for age, sex and intensity of noise exposure [23] were selected from the control group.

### DNA extractions

Peripheral blood (3 mL) was collected in ethylene diamine tetraacetic acid (EDTA) tubes and taken for DNA isolation and genotyping. DNA was extracted from blood samples of subjects by using the QIAcube HT and QIAamp 96 DNA QIAcube HT Kit (Qiagen, Dusseldorf, Germany) following the manufacturer’s instructions and stored at − 20 °C until use.

### SNP selection and genotyping

Target SNPs in the Notch1 genes were selected on the basis of the 1000 Genomes Project database and previous reports from the literature [24]. SNPs were selected from the National Center for Biotechnology Information (NCBI) database (http://www.ncbi.nlm.nih.gov/) with a minor allele frequency (MAF) > 0.10 in Han Chinese population. According to the criteria, twelve SNPs were identified. We then excluded SNPs which were in linkage disequilibrium (LD) (correlation coefficient R^2^ > 0.8 by Haploview 4.1 software). In the end, six candidate SNPs that fulfilled the criteria were selected (rs3124594, rs3124599, rs3124603, rs3125003, rs183200349, and rs575858778). Literature review indicated that rs3124594 and rs3124599 of Notch1 were reported in previous studies regarding lung cancer in northeast Chinese [21] and rs3124603 was selected as it was tagSNP.

The genotypes of the selected individuals at each polymorphic site were determined using ABI TaqMan SNP genotyping assays (Applied Biosystems, Foster City, CA, USA) and pre-designed commercial genotyping probes and primers. The extracted DNA and genotyping probes and primers were added to the TaqMan universal PCR master mix (Roche, Branchburg, NJ, USA) according to the manufacturer’s instructions. Genotyping was then performed on an ABI 7900 real-time PCR system (Applied Biosystems). The results were analyzed using the ABI 7900 system sequence detection software version 1.2.3 (Applied Biosystems).

### Statistical analyses

Statistical analyses were performed using SPSS 23.0 software (Chicago, IL, USA). Goodness-of-fit χ^2^ tests were conducted to determine whether the SNPs of the control subjects were in the Hardy-Weinberg equilibrium. Categorical variables were presented as percentages, and continuous variables were presented as the mean ± SD. Demographic and genotype information for NIHL cases and controls were compared using the Student’s t-test (for continuous variables) or χ^2^-test (for categorical variables). The associations between the genotypes of the three polymorphisms and risk of NIHL were estimated by computing odds ratio (ORs) and 95% confidence intervals (CIs) from unconditional logistic regression analysis with the adjustment for age, sex, as well as tobacco and alcohol consumption habits. Haplotype analysis was performed using the SHEsis platform [25]. All tests were two-sided and *P*-values were corrected (*P*c) by Bonferroni post test, and *P* < 0.05 was used to indicate statistical significance.

## Results

### Demographic characteristics of the study subjects and hardy-Weinberg test of selected SNPs

General demographic and life style features (age, sex, tobacco or alcohol consumption habits, duration of noise-exposed work time and noise intensity) and the high-frequency hearing threshold of the NIHL patients and controls are shown in Table 1. No significant differences between NIHL cases and controls were found regarding the general characteristics (*P* > 0.05). The average high-frequency hearing threshold was significantly higher in patients with NIHL (35.69 ± 9.92 dB) than for controls (14.01 ± 4.16 dB) (*P* < 0.001). General information about the selected SNPs and the results of the Hardy-Weinberg test are shown in Table 2. rs3124594, rs3124599 and rs3124603 are located in an intron variant region of Notch1 gene and all selected SNPs have minor allele frequencies ≥5% and are within the Hardy-Weinberg equilibrium (HWE) (*P* > 0.05).

**Table 1:** Demographic characteristics of study subjects

Variables	Cases (*n* = 535)		Controls (*n* = 535)		*P*
	n	%	n	%	
Age (years)					
Mean ± SD	40.40 ± 6.26		40.20 ± 5.79		0.595^a^
≤ 35	129	24.1	132	24.7	0.921^b^
35–45	303	56.6	305	57.0	
> 45	103	19.3	98	18.3	
Sex					
Male	496	92.7	496	92.7	1.000^b^
Female	39	7.3	39	7.3	
Tobacco use					
Now	311	58.1	301	56.3	0.476^b^
Ever	11	2.1	17	3.2	
Never	213	39.8	217	40.6	
Alcohol consumption					
Now	219	40.9	223	41.7	0.761^b^
Ever	9	1.7	12	2.2	
Never	307	57.4	300	56.1	
Duration of noise exposed work (years)					
Mean ± SD	18.54 ± 7.52		17.99 ± 6.99		0.216^a^
16	237	44.3	250	46.7	0.461^b^
> 16	298	55.7	285	53.3	
Noise exposure levels (dB)					
Mean ± SD	87.06 ± 7.69		87.46 ± 7.41		0.382^a^
85	243	45.4	232	44.4	0.392^b^
85–92	105	19.6	95	18.7	
> 92	187	35.0	208	36.9	
High frequency hearing thresholds (dB)					
Mean ± SD	35.69 ± 9.92		14.01 ± 4.16		< 0.001^a^
26	56	10.5	535	100.0	< 0.001^b^
> 26	479	89.5	0	0.0	

^a^*Students’* t-test

^b^Two-sided χ^2^ test

**Table 2:** General information of selected SNPs and Hardy-Weinberg test

SNP	Alleles	Chromosome	Functional Consequence	MAF		*P* for HWE ^b^
				Control	Database ^a^	
rs3124594	A/G	9:136501956	Intron variant	0.124	0.134	0.562
rs3124599	A/G	9:136509318	Intron variant	0.382	0.390	0.756
rs3124603	C/T	9:136515725	Intron variant	0.110	0.110	1.000

^a^Data from NCBI dbSNP

^b^*P* value of Hardy-Weinberg test

### Multivariate analyses of Notch1 SNPs with the risk of NIHL

Three Notch1 SNPs were genotyped in 1070 workers exposed to noise (535 NIHL patients and 535 controls). Table 3 shows the results of genotype and allele distributions of rs3124594, rs3124599 and rs3124603 in the codominant, dominant, recessive and allelic model. There were statistically significant differences in the genotype frequencies of rs3124594 and rs3124603 between cases and controls (*P* < 0.001 and *P* = 0.003, respectively) in the codominant model. In the dominant model, the rs3124594 GG and rs3124603 CT + TT were significantly associated with an increased risk of NIHL (*P* < 0.001 and *P* = 0.011 respectively). Subsequent logistic regression analysis adjusting for age, sex, alcohol and tobacco comsumption habits showed that individuals with rs3124594 GG and rs3124603 CT + TT had an increased risk of NIHL (OR = 2.45, 95% CI = 1.74–3.45, OR = 1.46, 95% CI = 1.09–1.94) compared with controls. In the recessive model, the rs3124594 GG + AG (OR = 4.19, 95% CI = 1.17–15.04) genotypes indicated a significantly increased risk for NIHL (*P* = 0.019). In the allelic model, the rs3124594 G (OR = 2.39, 95% CI = 1.74–3.29) allele indicated a significantly increased risk for NIHL (*P* < 0.001). Thus, our data revealed that the Notch1 SNPs rs3124594 and rs3124603 may have a significant association with an increased NIHL susceptibility.

**Table 3:** Distribution of three polymorphisms and the association with NIHL

Genetic models	Genotypes	Cases		Controls		*P* ^a^	Adjusted OR
		*n* = 535	%	*n* = 535	%		(95% CI)^b^
rs3124594							
Codominant	AA	3	0.6	12	2.2	< 0.001	1.00 (Ref.)
	AG	54	10.1	109	20.4		2.02(0.54–7.51)
	GG	478	89.3	414	77.4		4.70(1.31–16.87)
Dominant	AG/AA	57	107.0	121	22.6	< 0.001	1.00 (Ref.)
	GG	478	89.3	414	77.4		2.45(1.74–3.45)
Recessive	AA	3	0.6	12	2.2	0.019	1.00 (Ref.)
	GG/AG	532	99.4	523	97.8		4.19 (1.17–15.04)
Alleles	A	60	5.6	133	12.4	< 0.001	1.00 (Ref.)
	G	1010	94.4	937	87.6		2.39 (1.74–3.29)
rs3124599							
Codominant	GG	78	14.6	76	14.2	0.343	1.00 (Ref.)
	AG	234	43.7	257	48.0		0.88 (0.61–1.27)
	AA	223	41.7	202	37.8		1.07 (0.74–1.55)
Dominant	GG/AG	312	58.3	333	62.2	0.190	1.00 (Ref.)
	AA	223	41.7	202	37.8		1.18(0.92–1.51)
Recessive	GG	78	14.6	76	14.2	0.862	1.00 (Ref.)
	AA/AG	457	85.4	459	85.8		0.97 (0.69–1.36)
Alleles	G	390	36.4	409	38.2	0.396	1.00 (Ref.)
	A	680	63.6	661	61.8		1.08 (0.91–1.29)
rs3124603							
Codominant	CC	395	73.8	430	80.4	0.003	1.00 (Ref.)
	CT	134	25.0	92	17.2		0.51 (0.19–1.35)
	TT	6	1.1	13	2.4		1.59 (1.18–2.14)
Dominant	CC	395	73.8	430	80.4	0.011	1.00 (Ref.)
	CT/TT	140	26.2	105	19.6		1.46 (1.09–1.94)
Recessive	TT	6	1.1	13	2.4	0.105	1.00 (Ref.)
	CC/CT	529	98.9	522	97.6		2.17 (0.82–5.76)
Alleles	C	924	86.4	952	89.0	0.066	1.00 (Ref.)
	T	146	13.6	118	11.0		1.28 (0.99–1.66)

^a^Two-sided χ^2^ test

^b^Adjusted for age, sex, alcohol and tobacco consumption habits in logistic regression model

### Stratified analyses of rs3124594, rs3124599, and rs3124603 polymorphism and NIHL risk

The impact of the rs3124594, rs3124599, and rs3124603 genotypes in NIHL on several risk characteristics were analyzed using a recessive model. The results are shown in Table 4. In the group with noise exposure of ≤16 years, significant differences were found in the genotype distributions between cases and controls in rs3124594 (OR = 4.20, 95% CI = 2.44–7.24) and rs3124603 (OR = 1.73, 95% CI = 1.13–2.66). Individuals exposed to noise for > 16 years with the rs3124594 GG genotype had an increased NIHL risk (OR = 1.67, 95% CI = 1.06–2.63). Furthermore, workers exposed to noise levels of ≤85 dB with the rs3124603 CT/TT genotype, or noise levels of 85-92 dB and the rs3124599 AA genotype or noise levels of > 92 dB and the rs3124594 GG genotype had an increased risk of NIHL (OR = 2.06, 1.59 and 3.29 respectively).

**Table 4:** Stratified analysis of SNPs in a recessive model

SNPs	Group	Genotype	Duration of noise exposed work (years)		Expose level with noise (dB)		
			≤ 16	> 16	≤ 85	85–92	> 92
rs3124594	case	GG	217	261	212	96	170
		AG/AA	20	37	31	9	17
	control	GG	183	231	189	73	152
		AG/AA	67	54	43	22	56
	*P* ^a^		< 0.001	0.03	0.083	0.004	0.001
	Adjusted OR		4.20	1.67	1.55	3.29	3.65
	(95% CI)^b^		(2.44–7.24)	(1.06–2.63)	(0.94–2.57)	(1.40–7.71)	(2.03–6.57)
rs3124599	case	AA	98	125	97	40	86
		AG/GG	139	173	146	65	101
	control	AA	102	100	98	32	72
		AG/GG	148	185	134	63	136
	*P* ^a^		0.902	0.089	0.607	0.516	0.021
	Adjusted OR		1.05	1.36	0.91	1.29	1.59
	(95% CI)^b^		(0.73–1.51)	(0.97–1.90)	(0.63–1.32)	(0.71–2.33)	(1.06–2.39)
rs3124603	case	CT/TT	68	72	70	27	43
		CC	169	226	173	78	144
	control	CT/TT	47	58	38	21	46
		CC	203	227	194	74	162
	*P* ^a^		0.010	0.269	0.001	0.551	0.835
	Adjusted OR		1.73	1.24	2.06	1.19	1.08
	(95% CI)^b^		(1.13–2.66)	(0.84–1.84)	(1.32–3.22)	(0.61–2.31)	(0.67–1.75)

^a^Two-sided χ^2^ test

^b^Adjusted for age, sex, alcohol and tobacco use in a logistic regression model

### Association between the haplotypes of Notch1 SNPs with NIHL risk

Furthermore, haplotype frequency analysis of the three SNPs was performed between NIHL cases and controls (Table 5). Four common haplotypes (with a frequency of > 3%) derived from the three SNPs, accounting for 90% of the haplotype variations, were selected, and the other haplotypes were pooled in the mixed group. We found that the haplotype AAC (rs3124594-rs3124599-rs3124603) in the Notch1 gene was associated with an increased risk of NIHL (OR = 14.95), whereas the haplotype GGC and GGT were associated with a decreased risk for NIHL (OR = 0.11 and 0.02) compared with GAC.

**Table 5:** Frequencies of inferred haplotypes among the cases and controls and their association with risk of NIHL

Haplotypes ^a^	Case (*n* = 535*2)		Control (*n* = 535*2)		*P* ^b^	Adjusted OR	Global *P* ^c^
	n	%	n	%		(95% CI)	
GAC	787	73.6	398	37.2		1.00 (Ref.)	< 0.001
GGC	89	8.3	413	38.6	< 0.001	0.11 (0.08–0.14)	
GGT	7	0.7	221	20.7	< 0.001	0.02 (0.01–0.03)	
AAC	118	11.0	4	0.4	< 0.001	14.95 (5.48–40.79)	
Others ^d^	69	6.4	34	3.2	0.972	1.01(0.66–1.55)	

^a^The alleles of haplotypes were arrayed as rs3124594-rs3124599-rs3124603

^b^Two-sided χ^2^ test

^c^Generated by permutation test with 1000 times of simulation

^d^Haplotypes with a frequency < 0.03 (AAC/AAT/AGT/GAT) were pooled into the mixed group

### Comparison of the high-frequency hearing threshold shift in the three SNPs genotypes

**Fig. 1:**
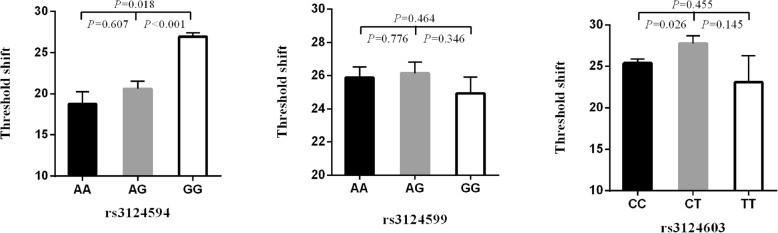
Comparison of high-frequency hearing threshold shift of all subjects. Comparison of high-frequency hearing threshold shift of rs3124594, rs3124599 and rs3124603 genotypes in all subjects. Data are presented as mean ± SE and analyzed by ANOVA

The high-frequency hearing threshold shift was compared in the rs3124594, rs3124599, and rs3124603 genotypes in 1070 noise exposed workers (Fig. 1). Individuals with the rs3124594 GG genotype had a significantly higher high-frequency hearing threshold shift than those with the AA or AG genotype (*P* = 0.018 and < 0.001). Individuals with the CT genotype of rs3124603 were also found to have a higher high-frequency hearing threshold shift than people with CC genotype (*P* = 0.026).

### Multifactor dimensionality reduction analysis of the interaction between the three SNPs

**Fig. 2:**
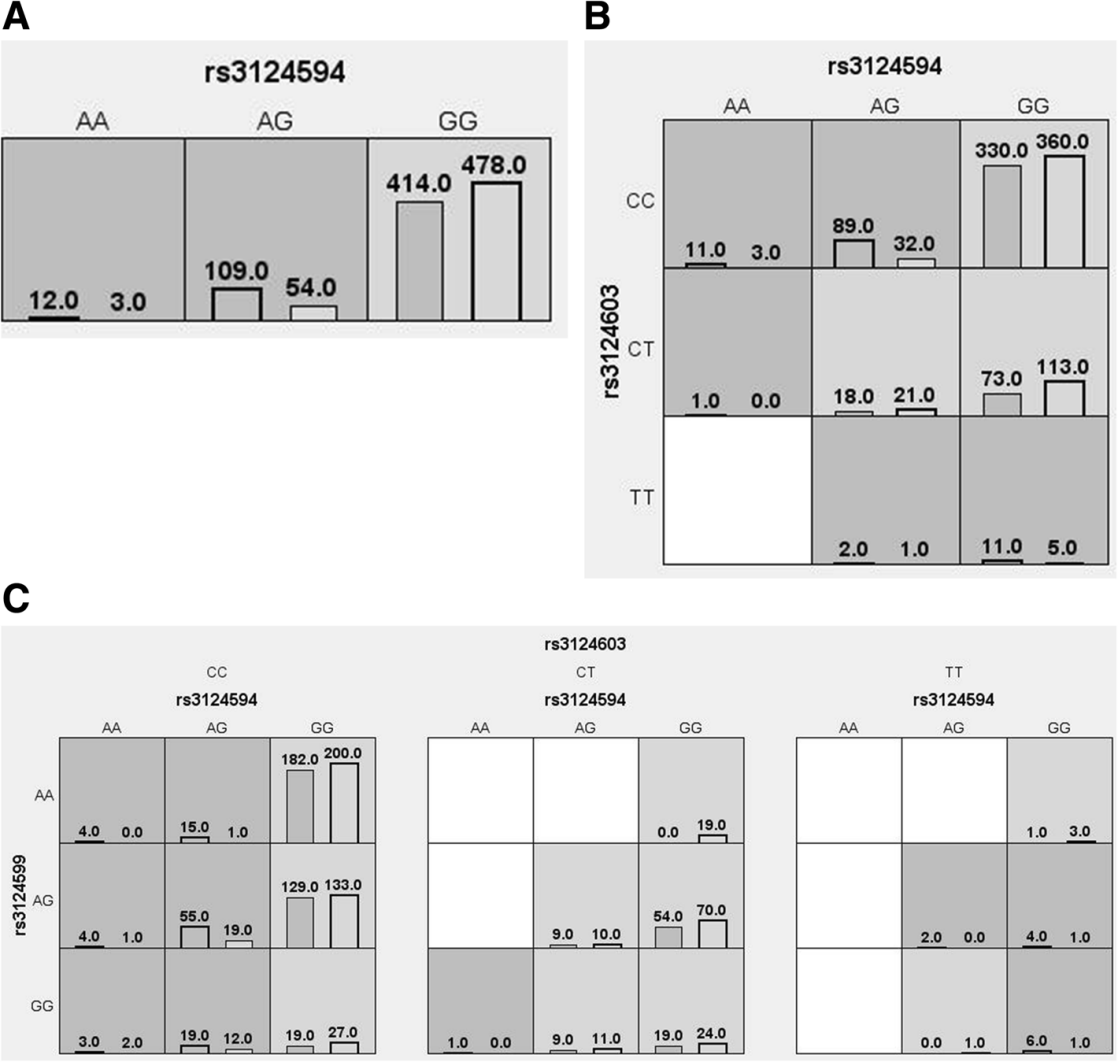
Graph model of the interaction between the three SNPs (**a**: rs3124594 model, **b**: rs3124594-rs3124603 model, **c**: rs3124594-rs3124599-rs3124603 model). Dark gray and light gray boxes presented the high- and low-risk factor combinations, respectively. Left bars within each box represented case while the right bars represented control. The heights of the bars are proportional to the sum of samples in each group

The results of the MDR analysis of the interaction between the three SNPs are shown in Table 6 and Fig. 2. The interaction results suggest that the rs3124594-rs3124603 and rs3124594-rs3124599-rs3124603 model was related to an increased risk of NIHL (OR = 3.26 and 3.60, *P* < 0.0001).

**Table 6:** MDR analysis results of the interaction between the three SNPs

Model	Training balanced accuracy	Testing balanced accuracy	Cross-validation consistency	*P*	OR(95%CI)
rs3124594	0.5598	0.5598	10/10	< 0.0001	2.45(1.74–3.45)
rs3124594-rs3124603	0.5683	0.5664	10/10	< 0.0001	3.26(2.23–4.77)
rs3124594-rs3124599-rs3124603	0.5722	0.5355	10/10	< 0.0001	3.60(2.43–5.34)

## Discussion

SNPs are a common type of genetic variation in the human genome, with about 15 million SNPs among all humans [26]. Haplotype is defined as a specific set of alleles on a single chromosome, or on a part of a chromosome, and has been an important factor in human genetics for decades [27]. Some haplotypes have a particular biological meaning such as the ones derived from SNPs located in the promoters, or the ones derived from non-synonymous SNPs. All these haplotypes are “subhaplotypes”. Using method of subhaplotyping can dramatically reduce the error rate on patient resolutions and haplotypes frequencies and minimise the risk of a false interpretation in genetic studies involving subhaplotypes [28]. The genomic distribution of SNPs is not homogenous; SNPs occur more frequently in the noncoding regions than in the coding regions of genes. At present SNPs are mostly detected by denaturing gradient gel electrophoresis, single-strand conformational polymorphism analysis, cleaved amplified polymorphic sequence assays, denaturing gradient gel electrophoresis or and allele-specific PCR (TaqMan SNP genotype-PCR).

In the present study, a genetic association analysis was performed on three selected Notch1 SNPs (rs3124594, rs3124599 and rs3124603) in 535 NIHL patients and 535 controls using the TaqMan SNP genotyping assay. We observed that the rs3124594 GG and rs3124603 CT + TT genotype in Notch1 were associated with a significantly higher risk of NIHL. Subsequent haplotype analysis showed that the haplotype AAC (rs3124594, rs3124599 and rs3124603) was associated with an increased risk of NIHL. These findings support our hypothesis that Notch1 polymorphisms may contribute to the susceptibility to NIHL. To the best of our knowledge, this is the first association study showing that the Notch1 gene is associated with an increased risk of NIHL in a Chinese population.

All of the three SNPs are located in a noncoding region of the Notch1 gene, which is in line with previous studies that have shown that SNPs in noncoding regions have functional consequences for the regulation of protein-coding genes and lncRNAs [29, 30]. The Notch pathway has a critical role in lateral inhibition, which regulates the cell fate determination in the cochlea [13]. During early embryonic development, inhibition of Notch/JAG2 and DLL1 has been shown to prolong the proliferation process of the prosensory cells in the inner ear [31]. Furthermore, neonatal mammalian cochlear supporting cells were found to have the ability to trans-differentiate into new hair cells when the Notch signaling pathway was blocked. It has been illustrated that, Notch signaling also acts as a negative regulator by inhibiting the proliferation of Lgr^5+^ progenitors and maintaining the homeostasis of cochlear sensory epithelium on cell numbers [18]. Taken together, this suggests that the noncoding SNPs rs3124594 and rs3124603 may have a significant role in the maintenance and repair of cells required for hearing through protein-coding genes or lncRNA regulation of the Notch pathway.

However, our study has several potential limitations. (*a*) The sample size of our study was relatively large compared to previous studies. However, due to the low biological effects of an individual SNP, the sample size may not have been sufficient for an appropriate statistical analysis. Therefore, further studies with increased sample size are needed to validate the effect of Notch1 polymorphisms on the risk of NIHL. (*b*) The study subjects of this case-control study were all Chinese Han people. Therefore our results may only apply to the Chinese Han population; (*c*) The selected SNPs are noncoding, which is why we assume that these variants are involved in the regulation of protein-coding genes and lncRNA regulation of Notch1.

## Conclusions

Here we provide the first piece of evidence illustrating an association between the polymorphisms rs3124594 and rs3124603 in the Notch1 gene and a significantly higher risk of NIHL. These noncoding SNPs may be involved in the regulation of protein-coding genes or of lncRNAs of Notch1. Thus, these findings suggest that the three Notch1 SNPs (rs3124594 and rs3124603) may play an important role in noise induced hearing loss and are potential new biomarkers for NIHL in noise exposed Chinese workers.

## Abbreviations

HC: Hair cells
MAF: Minor allele frequency
NIHL: Noise induced hearing loss
OR: Odds ratios
PTA: Pure-tone audiometry
SNP: Single nucleotide polymorphism
